# Psychological legacies of intergenerational trauma under South African apartheid: Prenatal stress predicts greater vulnerability to the psychological impacts of future stress exposure during late adolescence and early adulthood in Soweto, South Africa

**DOI:** 10.1111/jcpp.13672

**Published:** 2022-07-19

**Authors:** Andrew Wooyoung Kim, Rihlat Said Mohamed, Shane A. Norris, Linda M. Richter, Christopher W. Kuzawa

**Affiliations:** ^1^ Department of Anthropology University of California, Berkeley Berkeley CA USA; ^2^ SAMRC/Wits Developmental Pathways for Health Research Unit, Faculty of Health Sciences University of the Witwatersrand Johannesburg South Africa; ^3^ Global Health Research Institute, School of Human Development and Health University of Southampton Southampton UK; ^4^ DSI‐NRF Centre of Excellence in Human Development University of the Witwatersrand Johannesburg South Africa; ^5^ Department of Anthropology Northwestern University Evanston IL USA; ^6^ Institute for Policy Research Northwestern University Evanston IL USA

**Keywords:** Prenatal stress, psychiatric morbidity, stress sensitization, late adolescence, young adulthood, South Africa

## Abstract

**Background:**

South Africa's rates of psychiatric morbidity are among the highest in sub‐Saharan Africa and are foregrounded by the country's long history of political violence during apartheid. Growing evidence suggests that in utero stress exposure is a potent developmental risk factor for future mental illness risk, yet the extent to which the psychiatric effects of prenatal stress impact the next generation are unknown. We evaluate the intergenerational effects of prenatal stress experienced during apartheid on psychiatric morbidity among children at ages 17–18 and also assess the moderating effects of maternal age, social support, and past household adversity.

**Methods:**

Participants come from Birth‐to‐Twenty, a longitudinal birth cohort study in Soweto‐Johannesburg, South Africa's largest peri‐urban township which was the epicentre of violent repression and resistance during the final years of the apartheid regime. Pregnant women were prospectively enrolled in 1990 and completed questionnaires assessing social experiences, and their children's psychiatric morbidity were assessed at ages 17–18.

**Results:**

Full data were available from 304 mother–child pairs in 2007–8. Maternal prenatal stress in 1990 was not directly associated greater psychiatric morbidity during at ages 17–18. Maternal age and past household adversity moderated the intergenerational mental health effects of prenatal stress such that children born to younger mothers and late adolescent/young adult children experiencing greater household adversity exhibited worse psychiatric morbidity at ages 17–18. Social support did not buffer against the long‐term psychiatric impacts of prenatal stress.

**Conclusions:**

Greater prenatal stress from apartheid predicted adverse psychiatric outcomes among children born to younger mothers and adolescents/young adults who experienced greater concurrent stress. Our findings suggest that prenatal stress may affect adolescent mental health, have stress‐sensitising effects, and represent possible intergenerational effects of trauma experienced under apartheid in this sample.

## Introduction

South Africa's rates of mental, neurological, and substance use disorders are among the highest in sub‐Saharan Africa. In 2016, the estimated 12‐month prevalence for any psychiatric disorder was 16.2%, or approximately 9.1 million individuals (GBDCN, 2017). Despite these elevated rates of psychiatric morbidity, access to mental health treatment is poor: the treatment gap for mental disorders, epilepsy, and intellectual disability in South Africa is approximately 92% (Docrat, Besada, Cleary, Daviaud, & Lund, 2019). The current state of public mental health in South Africa is foregrounded by a long and recent history of institutionalised White supremacist policies implemented during the apartheid regime (c. 1948–1994). This period was characterised by systematic disenfranchisement of non‐White communities through various modes of social, economic, and political oppression (Beinart, 2001). Despite the legislative end of apartheid policies and the birth of a new democracy in 1994, South African society continues to be plagued by the persistent societal institutions of apartheid, including chronic poverty (Gibbs, Jewkes, Willan, & Washington, 2018), discrimination (Williams et al., 2008), and racialised class inequality (Adjaye‐Gbewonyo, Avendano, Subramanian, & Kawachi, 2016), all of which are known risk factors for psychiatric disorders (Allen, Balfour, Bell, & Marmot, 2014; Moomal et al., 2009; Myer et al., 2008).

In addition to these ongoing societal effects of poverty, structural violence, and inequality, there is growing evidence, from South African studies and other populations, that the stressors of the past could have lingering psychological effects that continue to influence socio‐emotional behaviour and mental health across the lifecourse. Growing evidence from the fetal origins of health and disease framework shows that past stress and trauma exposures, particularly those that occur during gestation, can increase future risk of psychiatric disease, such as externalising disorders (Betts, Williams, Najman, & Alati, 2015; MacKinnon, Kingsbury, Mahedy, Evans, & Colman, 2018; Rice et al., 2010) and internalising disorders (Betts et al., 2015; Davis et al., 2020; O'Donnell et al., 2014) in children and adolescents. While the majority of analyses on the intergenerational mental health effects of prenatal stress have come from samples in high‐income, Western contexts, a growing number of studies have detected intergenerational psychological signatures of maternal prenatal stress exposure in low‐ and middle‐income settings, such as samples in Brazil (Serpeloni et al., 2019), Ethiopia (Isaksson, Deyessa, Berhane, & Högberg, 2017), Nicaragua (Isaksson, Lindblad, Valladares, & Högberg, 2015), and South Africa (Ramchandani, Richter, Norris, & Stein, 2010). These studies have found direct relationships between greater levels of maternal prenatal stress and poor mental health among children and adolescents in the next generation.

The long‐term impacts of prenatal stress are hypothesised to shape adult psychiatric risk by altering the development and function of various stress regulatory mechanisms in humans, including the immune system, cardiovascular system, neurobiological function, neuroendocrine mechanisms, and psychological pathways (Barker, 1999; Gluckman & Hanson, 2004; Heim, Entringer, & Buss, 2019; Kuzawa & Kim, 2022; Taylor, 2010; Weinstock, 2008). Recent findings also suggest that these stress‐linked alterations in stress physiology may also affect the developing offspring through various pathways of genetic and non‐genetic inheritance, such as through the allelic variations in neurotransmission and neuroendocrine related genes, and epigenetic variation (McKenna, Hammen, & Brennan, 2021; Oberlander et al., 2008; Thayer, Wilson, Kim, & Jaeggi, 2018). While the specific biological mechanisms that underlie the long‐term psychiatric effects of prenatal stress are unknown, growing evidence from the literature on the fetal origins of psychopathology suggests that prenatal stress exposure alters the development, function, sensitivity of human stress physiological systems across the life course, which in turn may elevate one's risk for a psychopathological presentation and longer‐term disease. Prenatal stress‐linked alteration of stress physiological mechanisms has consistently been reported as both a prospective risk factor and cross‐sectional neuropsychiatric phenotype of later‐life psychiatric morbidity in the next generation (Davis et al., 2020; McKenna et al., 2021; Zohsel et al., 2014).

Recent evidence has suggested that prenatal stress exposure may also increase sensitisation to the effects of future postnatal stressors and elevate mental illness risk. The stress sensitisation hypothesis (Hammen, Henry, & Daley, 2000) proposes that the risk for adult mental illness following stressful life events is higher among individuals with a history of postnatal developmental trauma than among individuals without a history of developmental trauma. Growing research has extended this hypothesis to suggest that greater prenatal stress sensitises or potentiates future reactions to stress across infancy to young adulthood (Entringer, Khumsta, Hellhammer, Wadhwa, & Wust, 2009; Gutteling, de Weerth, & Buitelaar, 2005; Ping et al., 2020; Tollenaar, Beijers, Jansen, Riksen‐Walraven, & De Weerth, 2011), and early evidence shows that prenatal stress‐induced stress sensitisation is associated with elevated psychiatric disease risk in adolescents (Ping et al., 2020). Although the mechanisms of stress sensitisation are still unknown, early evidence suggest that prenatal stress may potentiate the future impacts of stress and trauma due to alterations in the reactivity of stress‐sensitive behavioural and physiological mechanisms, which may underlie the development of worse adult psychiatric outcomes (Hentges, Graham, Plamondon, Tough, & Madigan, 2019; Huizink & De Rooij, 2018). While an increasing number of studies are noting the long‐term impacts of prenatal stress on infant, child, and early/mid adolescent psychological status (Ilg et al., 2019; Koss & Gunnar, 2018; Ping et al., 2020; Zijlmans et al., 2015), few studies have examined the mental health impacts of prenatal stress into late adolescence and adulthood (Betts et al., 2015; Entringer et al., 2009) and also evaluated the potential stress sensitisation effects of prenatal stress (Koss & Gunnar, 2018; Ping et al., 2020; Zijlmans, Riksen‐Walraven, & de Weerth, 2015).

Finally, greater maternal social support, or the psychological and material resources provided by social relationships to help mothers cope with stress (Cohen & Wills, 1985), may buffer against the poor mental health effects of psychosocial stress (Cruwys et al., 2013). Social support may increase a mother's capacity to effectively process and overcome stressful experiences by providing temporary relief from taxing physical and social demands, preventing adverse health behaviours, and obtaining new resources, whether physical, financial, or emotional (Adamakos et al., 1986). Past research has found that maternal social support during pregnancy may also buffer against the potential long‐term psychological consequences of prenatal stress in the next generation (Graignic‐Philippe, Dayan, Chokron, Jacquet, & Tordjman, 2014; Takács, Štipl, Gartstein, Putnam, & Monk, 2021). The possible buffering effects of maternal prenatal social support against the long‐term psychological impacts of prenatal stress in the next generation, especially after infancy, is not well‐known.

This emerging evidence suggests that some proportion of the mental health burden of current populations could reflect the lingering biological imprint of past traumatic experience during gestation (Barker, 1999; Krontira, Cruceanu, & Binder, 2020; Kuzawa & Sweet, 2009). South Africa's recent history and the country's tumultuous transition into democracy raise the question of whether the traumas of apartheid may continue to have lasting effects, influencing risk for psychiatric morbidity across generations (Adonis, 2016; Kim, 2020; Stevens, Eagle, Kaminer, & Higson‐Smith, 2013). It furthermore raises the questions of which individuals face greater risks of the long‐term mental health impacts of prenatal stress and whether certain experiences or resources may buffer or protect against these effects, opening opportunities for targeted groups for public health interventions to reduce the societal burden of psychiatric morbidity (Heim et al., 2019). However, little work in South Africa has explored this hypothesis and its contribution to mental health. Furthermore, as the most current research is conducted in high‐income, Western contexts, it remains unclear if these effects are seen in non‐Western, low‐ and middle‐income contexts like South Africa, where adverse psychosocial and environmental conditions are more prevalent and where the burden of mental illness is substantially greater (Patel et al., 2007; Vigo, Thornicroft, & Atun, 2016).

This study examines the long‐term impacts of prenatal stress and trauma from South African apartheid on psychiatric morbidity during late adolescence/early adulthood. We also assess the role of younger maternal age as a potential risk factor for exacerbating the intergenerational mental health effects of trauma as well as the possible buffering role of social support against the long‐term effects of prenatal stress. And finally, we aim to test the stress sensitisation hypothesis by assessing whether prenatal stress potentiates the future psychiatric impacts of postnatal stress exposure during late adolescence and early adulthood. We hypothesise that: (a) greater prenatal stress will be associated with worse psychiatric morbidity at ages 17–18, (b) greater social support will buffer against the effects of prenatal stress and predict lower psychiatric morbidity, (c) the intergenerational mental health effects of prenatal stress will be greater among children born to younger mothers, and (d) prenatal stress will potentiate the psychiatric effects of later‐life adversity among children at ages 17–18. Adolescence is an important period in the development of future mental illness as most psychiatric conditions emerge during this stage (Breslau et al., 2017; Patel et al., 2007) and because adolescent psychiatric disease is a major risk factor for future adult mental illness (Pine, Cohen, Gurley, Brook, & Ma, 1998; Sawyer et al., 2012). The recent history of apartheid, high rates of mental illness, and low rates of healthcare access emphasise the need to elucidate possible mechanisms underlying the intergenerational mental health effects of prenatal trauma in order to improve public mental health in South Africa.

We include two operational definitions of ‘prenatal stress’ and ‘stressful life events’ here to clarify the meanings of these concepts in our analysis. ‘Prenatal stress’ refers to the collection of adverse events, psychosocial stress, and trauma that mothers faced during pregnancy in 1990. We also use ‘prenatal stress exposure’ or ‘intergenerational trauma’ in our manuscript in order to keep consistent with the larger literature on prenatal stress, which uses these terms interchangeably. ‘Stressful life events’ refers to the collection of adverse events, psychosocial stress, and trauma that individuals are exposed to across the lifecourse during postnatal development.

## Methods

### Study setting and participants

Data come from a longitudinal birth cohort in South Africa called Birth to Twenty Plus (Bt20+), which currently spans three generations of families in the greater Johannesburg‐Soweto area. The Birth to Twenty (Bt20+) study is both the largest and longest running longitudinal birth cohort study of child health and development in Africa. Bt20+ emerged as a collaboration between the University of the Witwatersrand in Johannesburg and the South African Medical Research Council with the aim to assess the impacts of rapid urbanisation towards the end of apartheid on the growth, health, well‐being, and educational progress of children. Soweto is the largest township outside of the city of Johannesburg and a site of immense cultural and historical significance in the struggle against apartheid. Racial and political violence, government divestment, and widespread protest were common in both cities, particularly at the legislative end of apartheid, which is when pregnant women were first recruited into the study.

All singleton live births delivered in public sector hospitals between April 23 to June 8, 1990 and who were residents in the metropolitan Johannesburg‐Soweto area six months after delivery were enrolled in Bt20+ (Richter, Norris, & Ginsburg, 2006). In late 1989, BT20+ began surveying pregnant women in public antenatal clinics to identify potential participants whose births would fall within the enrollment period. Enrolled Bt20+ neonates were cross‐checked with all government birth notifications during the 7‐week time period. The study area covered approximately 78 square miles at the time and included close to 3.5 million people, with about 400,000 informal housing units.

A total of 3,273 singleton children were enrolled in Bt20+ and 72% of the initial cohort continued to participate in the study after 17 years (Norris, Richter, & Fleetwood, 2007). The cohort is roughly representative of longer‐term residents of the metropolitan Soweto‐ Johannesburg region. Currently, the cohort underrepresents White children due to cohort enrollment taking place in public health facilities; White families were more likely to utilise private practitioners and facilities. Although Bt20+ children were all in utero during the apartheid regime, they became among the first generation born into a democratic South Africa, and colloquially known as ‘Mandela's Children’ because they were born shortly after Nelson Mandela's release from prison on February 11, 1990. Between 1990 and 2007, the scope of this analysis, Bt20+ families participated in 18 waves of data collection, and follow‐up studies continue. All participants provided assent and their parents provided written, informed consent. Ethical approval was obtained from the University of Witwatersrand Committee for Research on Human Subjects.

Antenatal surveys were conducted by seven trained, multilingual interviewers. The process of testing survey instruments before and during a data collection wave around the survey instruments were 3‐fold: (i) The survey instruments were piloted by the data collection team of experienced fieldworkers, nurses and coordinators – all local to the study area – using a convenient sample of non‐Bt20+ participants to examine the language and understanding; (ii) Following the pilot, an extensive debrief workshop was conducted whereby every single survey item was discussed, understandings clarified, challenging words/sentences/concepts translated into isiZulu and seSotho and verified, and then tested again with another convenient sample; and thereafter; (iii) Detailed Standard Operating Procedures, guidelines and translated material were drawn up, and the first 50 Bt20+ participants were seen as a study pilot group for finalisation of survey items, procedures and supporting data collection documents. All data collection staff were very familiar with the study setting and conversant in at least 4 languages. Through this process, we aimed to improve reliability and reduce interviewer influence/bias. The majority of surveys were conducted in antenatal services, with a quarter conducted at home. Zulu, Sotho and English were the most common languages used. Adolescents were surveyed in a research facility at the Chris Hani‐Baragwanath Hospital in Soweto and at home.

Before the full sample was achieved, 1,594 women were surveyed during their third trimester about their pregnancy, social experience, and household conditions. Of the initial 1,594 women, 1,051 women (65.9%) completed all of the remaining prenatal stress questions. In 2007, when the outcome measure was assessed among adolescents in the next generation, 407 of the initial 1,051 mothers participated in the follow up wave. The movement of individuals, name and address changes, and mortality were the main reasons for lost to follow‐up in this sample (Norris et al., 2007).

### Prenatal stress and social support

Prenatal stress exposure (G1) during the third trimester of pregnancy was collected using a 16‐item scale (α = 0.64), adapted from Bluen and Odesnik (1988) capturing information on exposure to various forms of family and community stresses, including police violence, injury, and incarceration. Yes/no responses indicated the presence or absence of each stressor during the previous 6 months. After exploratory analyses, two questions were dropped: one due to a low response rate (have you experienced any problems with your other children?) and the other due to contradictory concepts being assessed at once (domestic and familial violence vs. partner separation). Scores were then summed to create an overall measure of prenatal stress that ranged from 0 to 14.

Social support was measured using a series of four, yes/no questions to identify the absence or presence of instrumental and emotional support, including: people available to help, a confidante, being able to speak to her partner, belonging to a community organisation/church. All questions were summed to create a total score, which ranged from 0 to 4.

### Demographic, health, and socioeconomic variables

During the antenatal visit in 1990, expectant women were asked about their current housing situation (e.g. number of rooms, number of inhabitants), population group (i.e. ‘race’), marital status, age, and obstetric history. Gravidity was calculated from information on obstetric history and operationalised into a categorical yes/no variable (0 = no past pregnancy, 1 = past history of pregnancy). They were also asked whether they used tobacco or had been drinking alcohol during their pregnancy. At delivery, a series of questions about the neonate were administered to gather information about gender, birth order, birthweight, and gestational age. Birthweight (g) and gestational age (weeks) were obtained from the child's Road to Health Card, a patient‐held child medical record provided to all new mothers in South Africa.

Household socio‐economic status (SES) was assessed using an asset index which scored each participant according to the number of household physical assets that they possessed out of a possible 6 (e.g. television, refrigerator, washing machine, radio, telephone, home ownership, car). Household SES was measured again in 2006 using an updated list of assets (e.g. television, car, washing machine, refrigerator, phone, radio, microwave, cell phone, DVD, MNET, DSTV, computer, and internet). The asset index was designed based on standard measures used by the Demographic and Health Surveys (https://dhsprogram.com/), based on the work of Filmer and Pritchett (1999). To capture the overall SES environment during prenatal development and 2007, when the outcome measure was administered, an aggregate SES variable was constructed by summing standardised assets scores from both years of data collection.

### Household stressful life events

The occurrence of stressful life events in the household was assessed across two timescales, 6 months (i.e. “recent stress”) and 12 months (i.e. “past stress”), and reported by the caregiver of the index child based on yes/no responses. Thirteen life events were assessed and summed together to create a composite measure of household stress that occurred during the past 6 months (recent stress), which included death of a sibling, parent, and other family member (three separate questions), divorce, index child changing schools, a serious illness or hospitalisation experienced by the index child, caregiver, and family member (three separate questions), marital separation, increase in arguments with partner, caregiver separation from family for 2 weeks or more, a child leaving home, and unemployment. Additionally, six life events that occurred in the last year were summed to create a separate composite measure, which assessed whether any member of the household experienced robbery, harassment, threats, sexual molestation, and physical violence (past stress).

### General Health Questionnaire (GHQ‐28)

The General Health Questionnaire (GHQ‐28) is a 28‐item psychological screener that provides a measure of psychiatric morbidity based on four 7‐item scales: somatic symptoms, anxiety and insomnia, social dysfunction, and severe depression (Goldberg & Hillier, 1979). It assesses changes in mood, feelings, and behaviours during the past 4 weeks. The respondent evaluates their occurrence on a 4‐point Likert scale, ‘less than usual,’ ‘no more than usual,’ ‘rather more than usual,’ and ‘much more than usual.’ Seven questions are reverse scored and transformed before all responses summed. Bt20+ index children and their caregivers completed the GHQ during a follow‐up wave of data collection when the index children were 17–18 years old. The internal reliability of the GHQ‐28 in this sample was high (α = 0.91).

### Statistical analyses

All analyses were conducted using version 15.1 of Stata (Stata Corporation, College Station, TX, USA). Survey data were managed in the REDCap online management system. All variables were examined for outliers using descriptive statistics, visual tools including box and scatter plots, and percentage analysis. Bivariate analyses were conducted between prenatal stress, psychiatric morbidity at ages 17‐18, and covariates. Covariates were included based on a priori knowledge of social, biological, and obstetric risk factors that may potentially confound the relationship between prenatal stress and later‐life psychiatric morbidity (De Mola, De França, de Avila Quevedo, & Horta, 2014; Entringer et al., 2009; O'Donnell et al., 2013). The following variables were considered for inclusion as confounding factors: maternal age, maternal education, marital status, gravidity, tobacco use, and alcohol consumption during pregnancy, household density (ratio of the number of inhabitants to the number of rooms available for sleeping), social support, and child gender (*p* > .10). Adjusted birthweight and gestational age were also included as key covariates to examine the role of fetal growth restriction and preterm delivery as possible pathways by which prenatal stress affects late adolescent/early adult psychiatric morbidity. Adjusted birthweight was created by regressing birthweight on gestational age to create standardised residuals.

**Figure 1:**
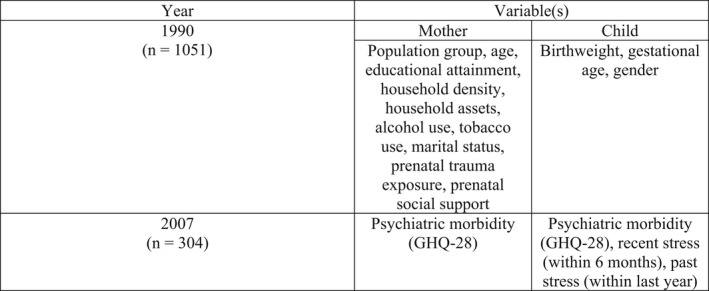
Timeline of study variables

With the exception of known confounding factors for the relationship between prenatal stress and later life psychiatric morbidity, only those that were statistically significant at the .1 level were included in the final models. These variables included infant gender, aggregate SES, gravidity, perceived social support during pregnancy, maternal education during pregnancy, household density, alcohol consumption during pregnancy, tobacco use during pregnancy, adjusted birthweight, gestational age, maternal age, recent stress, past stress, and maternal GHQ in 2007 (Figure 1). Multiple ordinary least squares (OLS) regressions were conducted to examine the impact of prenatal stress on late adolescent psychiatric morbidity.

The final analytical sample included 304 mothers and adolescents with requisite data (Table 1). Participants included in the analytical sample were similar to those excluded (*n* = 747) with respect to psychiatric morbidity at 17–18 years, prenatal stress, child gender, gravidity, alcohol consumption, tobacco use, social support, household density, SES in 2008, adjusted birthweight, recent stress, past stress, and maternal psychiatric morbidity (*p* > .05). Birthweight, gestational age, SES in 1990, maternal education in 1990, and the aggregate SES measure were significantly different from those excluded from the sample (*p* < .05). Children in the analytical sample exhibited higher birthweights and slightly longer gestations. Additionally, participants in the analytical sample reported lower household SES in 1990, lower aggregate SES levels, but more educated mothers in 1990. Thus, the analytic sample is somewhat disadvantaged compared with excluded group.

**Table 1:** Demographic characteristics, prenatal conditions, and psychiatric morbidity

Variables	Total (*n* = 304)		Female (*n* = 161)		Male (*n* = 143)	
	*N*	Mean	*N*	Mean	*N*	Mean
*Demographics*						
Population Group (‘Race’)						
Black	270		145		125	
Coloured	31		15		16	
Indian	3		1		2	
Maternal age (at enrollment)		25.5 (6.0)		25.3 (5.7)		25.6 (6.3)
Maternal educational attainment (% attended)						
No school or primary school	33		13		20	
Secondary school	244		136		108	
Professional/teaching/university	27		12		15	
Household density (people/room)		3.5 (1.7)		3.5 (1.9)		3.5 (1.6)
Household assets at 1990		3.9 (1.7)		3.9 (1.6)		3.9 (1.7)
Household assets at 2006		6.6 (2.4)		6.8 (2.4)		6.4 (2.4)
Maternal alcohol use in 1990						
Never	285		150		135	
Few times a year	11		6		6	
Once a month or more	8		5		2	
Maternal tobacco use in 1990						
Not at all	268		148		120	
Occasionally	13		5		8	
Daily	23		8		15	
Marital status						
Married/Partnered	90		39		51	
Single/Widowed	214		122		92	
Birthweight (g)		3149.2 (443.8)		3129.3 (447.2)		3171.7 (440.5)
Low birthweight (<2,500 g)	20	2262.3 (292.8)	11	2253.2 (385.2)	13	2343.1 (154.2)
Gestational age (weeks)		38.3 (1.2)		38.4 (1.2)		38.3 (1.3)
Preterm birth (<37 weeks)	17	35.5 (1.3)	8	35.5 (1.4)	9	35.4 (1.3)
*Psychological status*						
Maternal stressful events in 1990		2.4 (1.9)		2.4 (1.9)		2.5 (1.9)
Maternal social support in 1990		2.8 (0.9)		2.8 (0.8)		2.9 (1.0)
Child General Health Questionnaire (score)		45.2 (12.7)		47.9 (13.8)		42.1 (10.7)
High psychological risk (≥51)	74	63.3 (11.9)	55	63.2 (12.3)	20	63.1 (11.2)
Maternal General Health Questionnaire in 2007 (score)		47.5 (13.2)		45.5 (10.3)		49.8 (15.5)

## Results

In our sample of 304 mothers and adolescent pairs, the average number of traumatic events experienced within the past 6 months of the survey was 2.4/15 total events, while the average number of social support resources was 2.8/4 (Table 1). About 24% of the sample, or 74 adolescents, surpassed the cutoff (51/112) for high psychological morbidity based on their GHQ scores. Table 2 presents the results of the OLS regression analyses of fetal, maternal, behavioural, and environmental factors that predict psychiatric morbidity during late adolescence. The unadjusted model (Model 1) predicting GHQ scores on prenatal stress was positive although not significant (b = 0.53, *p* = .17, 95% CI, −0.22 to 1.3). Fully adjusted models found that prenatal stress was not significantly associated with psychiatric morbidity during late adolescent and early adulthood (Model 2). Gender was a significant predictor of psychiatric morbidity, where young men displayed lower mental health symptoms (b = −5.8, *p* < .001, 95% CI, −8.7 to −2.9). Gravidity, adjusted birthweight, and recent stress were significantly associated with psychiatric morbidity at the *p <* .1 level. Higher gravidity predicted lower psychiatric morbidity, while adjusted birthweight and recent stress were both directly associated with greater psychiatric morbidity.

**Table 2:** Multiple regression models of prenatal stress predicting adolescent psychiatric morbidity with covariates

	Model 1	Model 2
Prenatal stress (count)	0.5 ± 0.4	0.6 ± 0.4
Gender (male)		−5.8 ± 1.5***
Maternal age (year)		0.04 ± 0.2
Gravidity (yes/no)		−3.7 ± 1.9^†^
Adjusted birthweight^a^		0.003 ± 0.002^†^
Gestational age (week)		−0.2 ± 0.6
Alcohol (frequency)		−0.09 ± 0.9
Tobacco (frequency)		0.1 ± 1.4
Crowding (ratio)		−0.4 ± 0.4
Recent Stress (count)		1.5 ± 0.8^†^
Past Stress (count)		1.0 ± 1.2
Maternal GHQ (score)		−0.04 ± 0.06
Assets (count)		−0.2 ± 0.5
Maternal education (count)		−0.8 ± 0.9
Social support (count)		−0.9 ± 0.9
Intercept	43.9 ± 1.2***	62.9 ± 24.4*
Model *R* ^2^	0.0063	0.1071

	Model 3	Model 4	Model 5
Prenatal stress (count)	0.9 ± 1.3	4.8 ± 1.6**	0.1 ± 0.5
Prenatal stress × Social support	−0.1 ± 0.4		
Prenatal stress × Maternal age		−0.2 ± 0.1**	
Prenatal stress × Past Stress			1.2 ± 0.6*
Gender (male)	−5.8 ± 1.5	−5.7 ± 1.4***	−5.7 ± 1.5***
Maternal age (year)	0.1 ± 0.2	0.4 ± 0.2*	0.7 ± 0.2
Gravidity (yes/no)	−3.6 ± 1.9^†^	−3.6 ± 1.9^†^	−3.8 ± 1.9^†^
Adjusted birthweight^a^	0.003 ± 0.002^†^	0.003 ± 0.002*	0.003 ± 0.002^†^
Gestational age (week)	−0.2 ± 0.6	−0.1 ± 0.6	−0.2 ± 0.6
Alcohol (frequency)	−0.1 ± 0.9	−0.004 ± 0.9	−3.8 ± 1.9
Tobacco (frequency)	0.1 ± 1.4	0.1 ± 1.4	−0.1 ± 1.4
Crowding^b^ (ratio)	−0.4 ± 0.4	−0.3 ± 0.4	−0.3 ± 0.4
Recent Stress (count)	1.5 ± 0.8^†^	1.5 ± 0.8^†^	1.5 ± 0.8^†^
Past Stress (count)	1.0 ± 1.2	0.9 ± 1.2	−1.6 ± 1.7
Maternal GHQ (score)	−0.03 ± 0.06	−0.03 ± 0.1	−0.04 ± 0.06
Assets (count)	−0.2 ± 0.5	−0.3 ± 0.5	−0.1 ± 0.5
Maternal education (count)	−0.8 ± 0.9	−0.7 ± 0.9	−0.7 ± 0.9
Social support (count)	−0.7 ± 1.4	−1.1 ± 0.9	−0.9 ± 0.9
Intercept	62.5 ± 24.6*	50.9 ± 24.6*	61.7 ± 24.3*
Model *R* ^2^	0.1073	0.1288	0.1210

^a^ Birthweight adjusted for gestational age.

^b^ Number of household inhabitants/rooms used for sleeping.

^†^ *p* < .1 *p < .05 **p < .01 ***p < .001.

### Testing moderators of the prenatal stress‐later life psychiatric morbidity pathway

**Figure 2:**
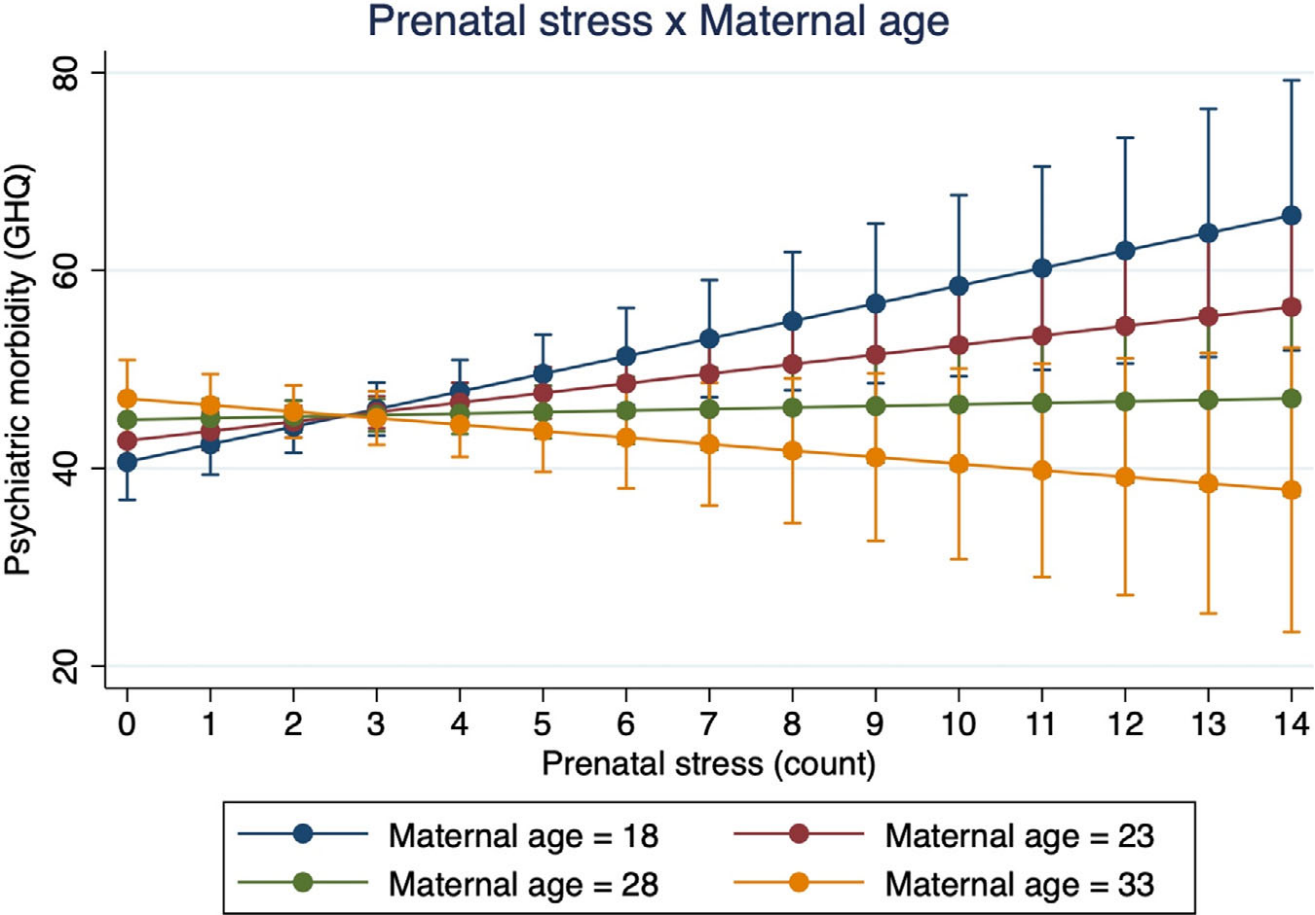
Interaction effect between prenatal stress and maternal age predicting psychiatric risk. This figure demonstrates the interaction effect between prenatal stress and maternal age predicting adolescent psychiatric risk at Year 17. The effect of prenatal stress on late adolescent psychiatric risk is much stronger among younger mothers (b = −0.17, *F*[1, 287] = 7.14, *p* = .008). There were significant main effects for prenatal stress and maternal age [Color figure can be viewed at wileyonlinelibrary.com]

**Figure 3:**
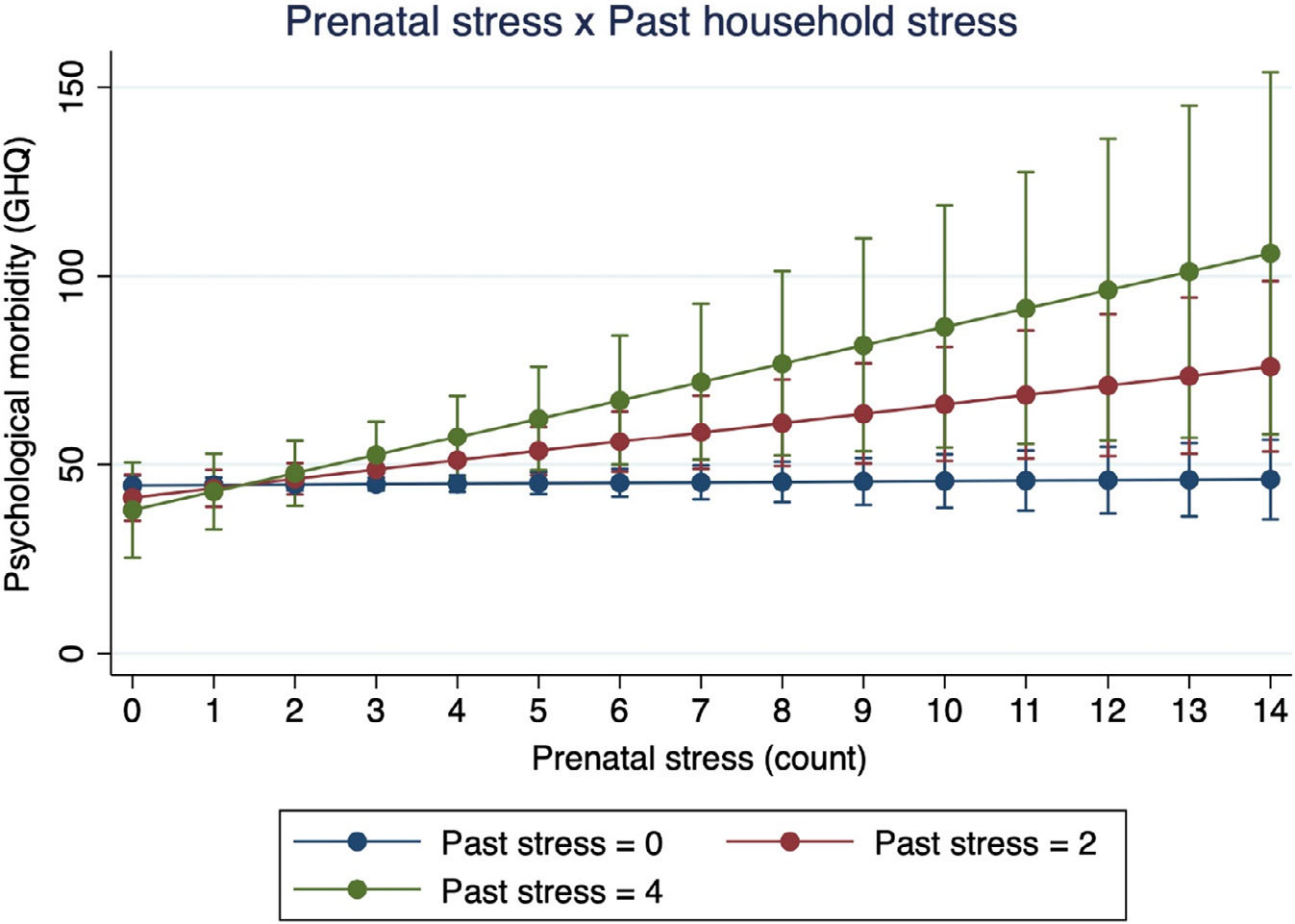
Interaction effect between prenatal stress and past household stress and trauma in the past year predicting psychiatric risk. This figure demonstrates the interaction effect between prenatal stress and past stress predicting adolescent psychiatric risk at Year 17. The effect of prenatal stress on late adolescent psychiatric risk is stronger as the degree of recent stress increases (b = 1.19, *F*[1, 287] = 4.52, *p* = .0343). There were no significant main effects for prenatal stress and recent stress. [Corrections made on 08 August 2022, after first online publication: Figure 3 has been replaced in this version to correct errors in the title and the legend.]

We next assessed potential moderators of the relationship between prenatal stress and future psychiatric morbidity in the index generation: social support, maternal age recent and past stress. To explore the possible effects of positive maternal social environments during pregnancy in ameliorating the long‐term mental health impacts of prenatal stress, we evaluated the effect of an interaction between social support and prenatal stress on psychiatric morbidity. Results reported a non‐significant interaction between social support and prenatal stress (Model 3: b = −0.092, *p* = .836, 95% CI, −0.97 to 0.79). Model 4 reports a negative and significant interaction between maternal age and prenatal stress severity (b = −0.17, *p* = .008, 95% CI, −0.29 to −0.044), showing that the adverse psychiatric effects of prenatal stress are stronger in children with younger mothers (Figure 2). Finally, to assess the stress‐sensitisation hypothesis, we evaluated the interaction between prenatal stress and both measures of household stress from the past 6 and 12 months. Our results reported a non‐significant interaction between prenatal stress and recent household stress from the past 6 months (b = −0.027, *p* = .951, 95% CI, −0.90 to 0.84) and a significant interaction between prenatal stress and past household stress from the past year (Model 5: b = 1.19, *p* = .0343, 95% CI, 0.089 to 2.3) (Figure 3). The effect of prenatal stress on late adolescent and early adult psychiatric morbidity was stronger in index children living in households with greater stress and trauma exposure.

## Discussion

In this longitudinal study of the intergenerational mental health impacts of prenatal stress in South Africa, we find that prenatal stress exposure during apartheid is not directly associated with greater psychiatric morbidity during late adolescence, 17–18 years after the timing of their fetal stress exposure. Social support did not moderate the association between prenatal stress and psychiatric outcomes. Maternal age and past household adversity, however, significantly moderated the intergenerational mental health effects of prenatal stress such that children born to younger mothers and late adolescent/young adult children experiencing greater household adversity in the past year exhibited worse psychiatric morbidity at ages 17–18. These patterns remained statistically significant after controlling for key demographic, social, and biological characteristics. Our findings provide early evidence that fetal development may be an important sensitive period for future psychiatric disease risk among children experiencing psychosocial stress during late adolescence and young adulthood. Our results also suggest that prenatal stress may sensitise children to the psychological sequelae of stress exposure later in life, representing one possible mechanism underlying the long‐term developmental effects of prenatal stress exposure. Overall, we find that prenatal stress is associated with greater vulnerability to the adverse psychological impacts of future stressors during late adolescence and young adulthood. These data shed light on the potential fetal origins of late adolescent mental health and the intergenerational effects of trauma from the period of apartheid in our birth cohort sample of South African mothers and children in Soweto‐Johannesburg. This study is among the first to prospectively assess the long‐term psychiatric impacts of prenatal stress into early adulthood in a low‐ and middle‐income country.

### Predictors of late adolescent/young adult psychiatric morbidity and moderators of the prenatal stress‐later life psychiatric morbidity pathway

The finding that greater prenatal stress is not related to future psychiatric morbidity is not consistent with the growing literature on the fetal origins of later life psychopathology, which generally reports a direct relationship between prenatal stress and later‐life psychiatric morbidity (Abbott, Gumusoglu, Bittle, Beversdorf, & Stevens, 2018; Bosch et al., 2012; Davis et al., 2020; O'Donnell et al., 2013; Ping et al., 2020). Retrospective and prospective studies of prenatal stress show that greater maternal social adversity during pregnancy predicts elevated risks for developing psychopathologies like depression, psychosis, and schizophrenia in the future (Lipner, Murphy, & Ellman, 2019; McQuaid, Darcey, Avalos, Fishbein, & VanMeter, 2019; Van den Bergh, Van Calster, Smits, Van Huffel, & Lagae, 2008). Studies examining the associations between maternal prenatal stress and psychiatric morbidity in late adolescence and early adulthood are very limited (Betts et al., 2015; Entringer et al., 2009), and the patterns remain inconclusive. The long‐term, intergenerational effects of prenatal stress, however, are significant when the moderating effects of past traumatic life events and maternal age on prenatal stress were independently assessed. We also find that female children faced greater psychiatric risk compared with males, and social support during pregnancy was not a significant moderator of the pathway between prenatal stress and later life psychiatric morbidity.

Female children exhibited elevated psychiatric risk relative to males in our sample, a pattern seen consistently across the literature and in our sample. During adolescence, young women across the world are twice as likely as men to develop depression (Salk, Hyde, & Abramson, 2017; Thapar, Collishaw, Pine, & Thapar, 2012) and other mental health conditions (De Vries, Davids, Mathews, & Aarø, 2018; Van Droogenbroeck, Spruyt, & Keppens, 2018; Wu et al., 2010). The stark elevation in female adolescent depression is understood to be influenced by a complex interplay between negative cognitive style, increased biological vulnerability, and negative affectivity that occur during puberty (Hyde et al., 2008). These interacting mechanisms of depressive vulnerability are further exacerbated due to stressful life events and broader societal conditions of gender‐based discrimination and violence. Past research in Bt20+ and South Africa corroborates the increased risk for depression and other mental health conditions in young women (Fernander et al., 2006; Meehan, Peirson, & Fridjhon, 2007; Richter, Ahun, Besharati, Naicker, & Orri, 2021; Sui et al., 2021).

Our findings on the interaction between prenatal stress and younger maternal age corroborate past research, which highlights the numerous socioeconomic, gendered, and social adversities faced by younger pregnant mothers in Soweto and other communities in South Africa (Makola, 2011; Macleod, 1999; Richter et al., 2006; Willan, 2013). Young motherhood in Soweto is understood to be a highly stigmatised and morally compromised status and a period of greater vulnerability to certain forms of violence in the township and other communities in South Africa (Panday, Makiwane, Ranchod, & Letsoalo, 2009). Past research in Bt20+, Soweto, and elsewhere shows that young mothers frequently receive disappointment and negative attitudes from their parents, ridicule and shame from nurses, and stigma from community members (Macleod, 1999; Makola, 2011; Richter et al., 2006; Willan, 2013). Past studies have reported that infants of young teenage mothers were lighter at birth relative to neonates from older mothers (Cameron, Richter, McIntyre, Dhlamini, & Garstang, 1996; Fraser, Brockert, & Ward, 1995; Rothberg, Shuenyane, Lits, & Strebel, 1991). The long‐term developmental effects of lower birthweight are well‐documented – lower birthweight is associated with increased risk for a wide range of adolescent and adult mental illness risk across the lifecourse (Abel et al., 2010; Barker, 2004; Lærum et al., 2019; Orri et al., 2019).

Results also show that adjusted birthweight, but not gestational age, is an important predictor of later life psychiatric morbidity in this sample. Interestingly, adjusted birthweight was positively and significantly related to late adolescent/early adulthood psychiatric morbidity. Our finding, however, that adjusted birthweight strengthens the coefficient on prenatal stress after inclusion into the model provides preliminary evidence that fetal growth may not be involved in the relationship between prenatal stress and adolescent psychiatric morbidity. The direct effect of adjusted birthweight on psychiatric morbidity at 18, however, conflicts with past literature on the fetal origins hypothesis that suggests that adverse intrauterine exposures and lower birthweight, potentially due to stress‐induced alterations in gestational neuroendocrine activity, can durably impact health, development, and disease risk in the next generation (Kuzawa, 2008; Monk, Lugo‐Candelas, & Trumpff, 2019; O’Donnell & Meaney, 2017). While we are unsure of the reasons underlying the direct relationship between adjusted birthweight and later‐life psychiatric risk, a small handful of studies have found similar associations between heavier birthweights (among non‐macrosomic babies) and greater psychiatric risk in the future (Gunnell, Rasmussen, Fouskakis, Tynelius, & Harrison, 2003; van Mil et al., 2015; Wegelius et al., 2013). Past studies have shown sex differences in fetal growth rates and preterm birth with boys typically showing higher incidence of adverse birth outcomes (Di Renzo, Rosati, Sarti, Cruciani, & Cutuli, 2007), yet there were no significant sex differences in gestational age nor birthweights in this sample.

Finally, we find that perceived social support during pregnancy did not buffer against the later‐life effects of trauma exposure during pregnancy. Prenatal social support did not appear to have an independent protective effect on adolescent psychiatric morbidity, yet the presence of a partner during pregnancy did significant predict lower adolescent GHQ scores. The insignificant effect of prenatal social support on future psychiatric morbidity, either from a direct protective effect on adolescent mental health or buffering against the psychiatric impacts of prenatal stress, may be attributed to the limited strength of our social support measure, which primarily assessed interpersonal support and a single, dichotomous measure of group membership.

Psychologists have emphasised the importance of assessing the degree, frequency, and timing, and modes of social support, similar to the impacts of stress and trauma, when examining psychosocial and physiological impacts (Dunkel Schetter, 2011; Orr, 2004). Past studies report protective effects of prenatal social support on postnatal outcomes, including better infant birth outcomes (Feldman, Dunkel‐Schetter, Sandman, & Wadhwa, 2000; Orr, 2004) and lower child adiposity (Katzow, Messito, Mendelsohn, Scott, & Gross, 2019). Prenatal social support, in the form of a presence of a partner during pregnancy, has also shown to buffer the adverse effects of prenatal social adversity to predict better cognitive and psychiatric outcomes in children (Spann, Bansal, Hao, Rosen, & Peterson, 2020). Finally, early evidence suggest that prenatal social support may buffer against the impacts of maternal stress and contribute to healthier cortisol regulation (Field, Diego, Delgado, & Medina, 2013; Luecken et al., 2013). Further research is necessary to identify both the protective and buffering effects of prenatal stress to ameliorate the long‐term disease outcomes of intergenerational stress and trauma.

### Evidence for prenatal stress‐linked stress sensitisation in late adolescence/early adulthood

Our data show that recent stress from the past 6 months was a positive and marginally significant predictor of psychiatric morbidity at 17–18, while the effect of traumatic life experiences from the past 12 months was only significant when interacted with prenatal stress. The significant interaction between prenatal stress and past year traumatic experiences, showing that the direct relationship between prenatal stress and psychiatric morbidity is stronger in children with greater household traumatic events, provides supporting evidence for the stress sensitisation hypothesis (Hammen et al., 2000; McLaughlin, Conron, Koenen, & Gilman, 2010; Van den Bergh et al., 2008). In our study, it is only after interacting with elevated levels of past stress that the long‐term sensitising effects of prenatal stress on later life psychiatric morbidity become apparent.

The stress sensitising effect of prenatal stress may only significantly interact with past stress (from the past 12 months) trauma rather than with recent stress (from the past 6 months) because of the nature of the experiences queried by each measure. Reported by the mother during the survey, our composite measure of past stress consisted of six questions assessing a collection of objective traumatic events that are more likely to have household‐level impacts specifically on the index child compared with the events assessed in the recent stress variable. Also reported by the mother, our measure of recent stress may also query household experiences that may have marginal to no impact on the index child's psychiatric morbidity. These experiences include unemployment, divorce, or death of a relative. Nevertheless, our data show interesting stress sensitisation effects due to the interaction between prenatal stress and household stress exposure from the past year.

Our findings advance the literature on stress sensitisation in a number of ways. First, we find that prenatal stress may be an important factor in shaping postnatal stress sensitivity up to 18 years after birth. A large portion of studies that report evidence for stress sensitisation have identified long‐term effects due to stress and trauma during childhood (Heim et al., 2019; Kendler, Kuhn, & Prescott, 2004; McLaughlin et al., 2010). A small yet growing number of studies have identified stress sensitisation effects associated with prenatal stress (Entringer et al., 2009; Gutteling et al., 2005; Ping et al., 2020). Second, our results report evidence for greater prenatal stress‐linked stress vulnerability in late adolescence and early adulthood, 17–18 years after fetal exposure to maternal stress. This finding suggests that the stress sensitising effects of prenatal stress may extend further into the lifecourse, as most studies on prenatal stress‐linked stress sensitisation have identified outcomes among infants, children, and young adolescents. One exception is a study by Entringer et al. (2009) who identified associations between retrospectively reported experiences of maternal prenatal stress and neuroendocrine stress reactivity in young adult offspring.

### Potential mechanisms underlying prenatal stress‐linked stress sensitisation and psychiatric outcomes in late adolescence/early adulthood

Given these data and existing findings on the fetal origins of late adolescent and early adult psychopathology, there are two possible developmental mechanisms that may facilitate the lasting impacts of prenatal stress on future psychiatric morbidity. First, greater histories of prenatal stress may increase the severity of behavioural and psychiatric conditions that increase emotional and biological sensitisation to future stressors and adverse events, such as depression, anxiety, and other mood disorders. Additionally, the major symptoms of depression, such as persistent feelings of victimisation, learned hopelessness and helplessness, and negative appraisal (Folkman, Lazarus, Gruen, & DeLongis, 1986; Peterson & Seligman, 1983) may have elevated individual sensitivity to and appraisal of recent stressful events (Medrano & Hatch, 2005; Peterson & Seligman, 1983) and neuroendocrine sensitisation (Stroud, Davila, Hammen, & Vrshek‐Schallhorn, 2011) to future stressors. Thus, greater sensitivity to and appraisal of past stress may have emerged as a function of the long‐term depressive and psychological effects of prenatal stress.

Second, increased severity of prenatal stress may cause durable increases in psychological and physiological stress reactivity (e.g. HPA axis, the immune system, and brain function) into adulthood, which may make individuals respond worse to future stressors, and in turn increase one's risk of developing a psychopathology (Hammen et al., 2000; Heim et al., 2019; Kendler et al., 2004; McLaughlin et al., 2010). In one study, researchers found that women who experienced greater maternal anxiety during pregnancy were more likely to have children with flattened diurnal cortisol slopes, which predicted depression in female adolescents (Van den Bergh et al., 2008). The growing literature on the long‐term health effects of prenatal stress is consistent with the larger body of scholarship on the effects of postnatal early life stress, which are known to have similar lasting effects on neuroendocrine, inflammatory, and molecular mechanisms across development, extending into adulthood (Gustafsson, Janlert, Theorell, & Hammarström, 2010; Heim & Binder, 2012; Taylor, 2010). While we cannot completely rule out the potential role of certain stress physiological mechanisms in driving the prenatal stress‐late adolescence relationship without directly assessing the system (e.g. salivary cortisol, cytokines, etc.), multiple stress‐sensitive biological pathways likely contribute to elevations in psychiatric risk at the same time.

Another possibility is that prenatal stress may be indicative of larger and longer‐term socioeconomic patterns of mothers and children in Soweto. Substantial evidence illustrates the durable impacts of poverty and material deprivation during pregnancy. Chronically low socioeconomic status between gestation and adulthood predicted flatter diurnal cortisol rhythms in a large sample of Filipino adults (Desantis, Kuzawa, & Adam, 2015). Future longitudinal research is needed to determine the underlying stress physiological mechanisms by which prenatal stress influences future stress sensitivity and psychopathological morbidity.

In many past published studies of the lifecourse impacts of prenatal stress, the source of maternal social adversity stem from violent and oppressive conditions linked with political turmoil, war, famine, and numerous forms of social inequality (Kertes et al., 2016; Kim, Nyengerai, & Mendenhall, 2022; Roseboom, de Rooij, & Painter, 2006; Yehuda et al., 2016). Despite the formal end to these forms of political violence, the developmental and health consequences of trauma exposures among pregnant women can durably extend across the lifecourse of the future child and may even impact the subsequent generation (Barbarin & Richter, 2013; Kuzawa & Sweet, 2009). These developmental pathways and health consequences of embodied trauma may represent mechanisms that drive mental health inequalities and disproportionately impact marginalised populations with high levels of stress and trauma exposure. Inasmuch as scientists aim to reverse the past impacts of embodied trauma and social oppression, the ongoing legacies of colonialism, structural violence, and historical traumas, such as apartheid, must be recognised and addressed to prevent future mental health inequities from emerging.

## Limitations

Our study is not without limitations. Unfortunately, the timeframe of the queried stress exposure in the prenatal stress measurement does not specify the exact moment of exposure as the participants were asked to report stressors that occurred in the past six months. Women completed the antenatal stress questionnaire during their third trimester, meaning that the reported stressor could have occurred anytime between the first and third trimester. Future research will benefit from knowing the exact periods of prenatal stress and stress physiological function to understand the potential developmental and physiological effects on the child. While we take advantage of data from a prospectively designed longitudinal study, our sampling strategy may have introduced bias into our analysis due to attrition and the missingness of data. Also, our single measure of mental health prevented us from identifying individuals with chronically high levels of psychiatric morbidity, which may have biased the estimates of our associations away from the null. We also did not record the language used to conduct the survey interviews in the 2007 data collection wave when psychiatric morbidity was assessed, which may have introduced biased due to issues of cultural meaning and linguistic translation of mental health experiences. Additional maternal influences during the child's development between birth and 17 years old which were unmeasured in this analysis may have also shaped the child's psychiatric morbidity at age 17.

Additionally, our measures of recent stress from the past six months, stressful life events from the past year, and prenatal social support did not exceed the acceptable level of internal reliability (α = 0.6). The surveys assessing recent and past stress also queried different sets of stressors, which limited our ability to identify whether the timing or content of postnatal stress affected our stress sensitisation and psychiatric outcomes. Because these two stress measures were endorsed by the mother rather than the index child, the degree to which these stressors affected the index child is unknown. However, the composite measure of past stress assessed a collection of objective traumatic and stressful life events that were more likely to have household‐level impacts on the index child compared with the events assessed in the recent stress variable, which may explain why we detected stress sensitisation effects as a result of our measure of past, rather than recent, stress. Notwithstanding, these stressful life events were vital to consider in our secondary data analysis given the normalcy of social adversity and interpersonal violence in our sample at the time of data collection.

## Conclusions

In this analysis of the intergenerational mental health impacts of prenatal stress in a large birth cohort in Soweto, South Africa, we find that prenatal stress is associated with greater vulnerability to the adverse psychological impacts of future stressors during late adolescence and young adulthood, 17‐18 years after fetal exposure to maternal stress. Our data show that greater prenatal stress from apartheid predicted adverse psychiatric outcomes among children born to younger mothers and adolescents and young adults who experienced greater household stress in the past year. These findings suggest that greater prenatal stress may adversely affect late adolescent and young adult mental health, have stress‐sensitising effects in children, and represent possible intergenerational effects of trauma experienced under apartheid in this sample of South African mothers and children in Soweto.
